# Supercritical Impregnation of PLA Filaments with Mango Leaf Extract to Manufacture Functionalized Biomedical Devices by 3D Printing

**DOI:** 10.3390/polym13132125

**Published:** 2021-06-28

**Authors:** José María Rosales, Cristina Cejudo, Lidia Verano, Lourdes Casas, Casimiro Mantell, Enrique José Martínez de la Ossa

**Affiliations:** Chemical Engineering and Food Technology Department, Wine and Agrifood Research Institute (IVAGRO), University of Cadiz, Puerto Real, 11519 Cadiz, Spain; jose.rosalessanchez@alum.uca.es (J.M.R.); lidia.veranonaranjo@alum.uca.es (L.V.); lourdes.casas@uca.es (L.C.); casimiro.mantell@uca.es (C.M.); enrique.martinezdelaossa@uca.es (E.J.M.d.l.O.)

**Keywords:** supercritical, polylactic acid, impregnation, biomedicine, medical device, mangifera indica, 3D printing, additive manufacturing

## Abstract

Polylactic Acid (PLA) filaments impregnated with ethanolic mango leaves extract (MLE) with pharmacological properties were obtained by supercritical impregnation. The effects of pressure, temperature and amount of extract on the response variables, i.e., swelling, extract loading and bioactivity of the PLA filaments, were determined. The analysis of the filaments biocapacities revealed that impregnated PLA filaments showed 11.07% antidenaturant capacity and 88.13% antioxidant activity, which after a 9-day incubation shifted to 30.10% and 9.90%, respectively. Subsequently, the same tests were conducted on printed samples. Before their incubation, the printed samples showed 79.09% antioxidant activity and no antidenaturant capacity was detected. However, after their incubation, the antioxidant activity went down to only 2.50%, while the antidenaturant capacity raised up to 23.50%. The persistence of the bioactive properties after printing opens the possibility of using the functionalized PLA filaments as the feed for a three-dimensional (3D) printer.

## 1. Introduction

Additive manufacturing, and specially its most popular type, fused deposition printing, commonly known as three-dimensional (3D) printing, has recently shot up. Many research studies from different fields have proven the enormous efficiency of this production method over other conventional techniques, since it reduces production time and investment demands [1]. Moreover, it is in the healthcare industry where it has experienced its most prominent increment. Advanced scanning technologies for medical use and widespread application of computer-aided designs have contributed to the engineering of more accurate biomedical devices, such as endoprostheses or cell seeding scaffolds, whose geometries could result very intricate for more traditional manufacturing procedures [1,2,3]. Additionally, when the aforementioned technologies have been combined with 3D printing, pre-surgical planning and surgery have been improved and favored patient recovery [4]. For example, metal stents are manufactured by cutting out material from a hollow cylinder by means of a high precision laser to produce the desired shape and structure. 3D printing has appeared as a more rapid and affordable alternative to the traditional laser cutting process [3,5]. Furthermore, 3D allows a more customized manufacturing of the medical devices to fit exactly each patient’s needs. According to the research carried out by Guerra et al. [6,7], both PLA and Polycaprolactone (PCL) are suitable polymers for stent manufacturing, since they are fully degradable through the hydrolysis of their ester bounds, even if they still provide an excellent durability [8]. Nevertheless, the use of polymers as manufacturing material for biomedical devices also presented some drawbacks with regard to biocompatibility. Thus, underperformance or the proliferation of certain cells [9] could represent a hazard for the patient. On the other hand, in the specific case of PLA, due to its hydrophobicity, it is specially difficult for cells to become attached onto its surface [10]. Therefore, given that polymers require to be properly functionalized to make sure that they fulfill the function they are designed for [10,11,12], the particular process that is used to integrate the pharmaceutical agents into the biodegradable polymer device is of utmost importance and must therefore be selected and designed with maximum care. Some thermal procedures, where the device is submerged into a specific drug solution or partially fused to get it mixed with the active substance, may pose a risk to the chemical structure of the drug. Besides, an additional production step is often required to remove the solvent, which negatively affects the efficiency of the whole process [13].

On the contrary, Supercritical Technologies (SCT), have displayed a number of advantages. Not only they are environmentally friendly, but they do not seem to affect the integrity of a large number of labile substances. Thus, thanks to its particular features, supercritical technology allows the production of solvents whose density is similar to that of its liquid state but being more compressible. This particular property also means that solvent’s density can be modified according to pressure, which means that—since the solute’s solubility level is proportional to the solvent’s density—SCT can provide not only a high solution capacity, but also a high selectivity [14,15]. Thus, this technology can be used to impregnate polymers with a large number of agents in order to confer their capabilities to the polymeric matrices. This process known as Supercritical Solvent Impregnation (SSI) has already been described as a safe and clean procedure that can be carried out in a single step [16,17,18].

As mentioned above, it is also highly selective and, by modulating some parameters such as pressure, temperature or time, it allows a precise control of both the sort and amount of compounds to be impregnated [13,17,19] while preserving their integrity [16,17]. In terms of supercritical solvents, carbon dioxide has shown to be rather efficient, because of its moderate critical point (31.1 °C and 73.8 bar), low price and high availability. Moreover, it is neither toxic nor inflammable, as well as almost inert [13]. The main disadvantage of CO${}_{2}$ is its low polarity, which can be modulated by adding a co-solvent, such as ethanol. As it has been outlined above, SSI can generate bioactive polymers that have the capacity to perform as pharmaceutical devices after shaping the polymer into a functional structure. The polymeric device capabilities will entirely depend on the impregnated bioactive agent, which will determine the type of therapeutic action to be expected from the device. Natural agents can be obtained from a wide range of sources, and agro-industrial wastes appear as an important one. In this sense, mango (*Mangifera indica* L.) as an agricultural product of large global production generates immense amounts of waste [20,21,22,23].

An extensive range of compounds found in *Mangifera indica* L., leaves, seeds or even tree bark have been extensively used in traditional medicine for the treatment of different diseases [20,24,25,26]. One of the most relevant compounds in mango leaves is mangiferin, not only for its high proportion, but also for its antioxidant and anti-inflammatory capacities [26,27,28], which have been endorsed by both in vitro and in vivo experiments [29,30,31]. For instance, mangiferin has exhibited anti-inflammatory responses against different inflammation mechanisms, such as the inhibition of nitrogen monoxide production, which cause blood vessels dilatation [30], or the inhibition of some pro-inflammatory factors such as interleukin-1β (IL-1β) [32,33,34], or tumor necrosis factor (TNF-α) [31,35], both of which play an important role during the acute phase of inflammation processes. In addition, a recent research work carried out by Jiang et al. [22] concluded that mangiferin also shows a cardioprotective capacity against cardiomyocyte apoptosis caused during heart failure.

Although the supercritical impregnation of a polymer with a natural extract, such as olive leaf extract, has been previously reported [19], the usage of a bioactive impregnated polymer filament to feed a 3D printer is a whole new conception. Bioactive polymeric devices have been previously designed as films to be used as patches without any further transformation, while in our study, impregnated PLA filaments were fused using a 3D printer and modeled in order to produce biomedical functionalized devices. To accomplish this objective, the effect of the impregnation process variables, i.e., temperature, pressure, and amount of extract added into the reactor on extract loadings and filament integrity have been determined. Since 3D printing is a thermal process in which the polymer is exposed to high temperatures, it is essential to establish a comparison between the bioactive properties of the impregnated polymeric samples against those of the final device produced by means of the 3D printer.

## 2. Materials and Methods

### 2.1. Chemical Reagents and Raw Material

For the impregnation process, 1.6 mm diameter Polylactic acid (PLA) filaments were purchased from Mundo Reader S.L. (Madrid, Spain). The polymeric material was 100% PLA white-color (1.24 g/cm${}^{3}$) with no additives. Filament’s thermal properties are described in Table 1. The *M. indica* L. leaves were provided by the Institute for subtropical and Mediterranean horticulture “La Mayora” (Malaga, Spain) and used to elaborate the bioactive extract.

The carbon dioxide (99.99%) used in supercritical impregnation was acquired from Abello Linde S.A. (Barcelona, Spain). The reagents for phosphate-buffered saline (PBS) formulation were: NaCl, KCl, Na${}_{2}$HPO${}_{4}$, and KH${}_{2}$PO${}_{4}$. Anhydrous acetic acid was used to adjust the pH of PBS. Ethanol (96%) was used to obtain *M. indica* L. extract. All these reagents were supplied by Panreac AppliChem (Darmstadt, Germany). 2,2-diphenyl-1-picrylhydrazyl (DPPH) was used for antioxidant assay, and purchased from Sigma-Aldrich (Steinheim, Germany). The dehydrated ovoalbumin, which was supplied by Agrovin (Ciudad Real, Spain), was used in the protein denaturation inhibition assay.

### 2.2. Extraction

The mango leave extracts (MLE) were obtained by the Pressurized Liquid Extraction method (PLE). Two extraction runs were conducted by means of a supercritical extraction equipment provided by Thar Technologies (model SF1000, Pittsburgh, PA, USA). A description of the equipment and the procedure has been published in a previous work, where the extraction conditions of mango leaves have been optimized [36,37]. For each extraction, 500 g of *Mangifera indica* L. leaves were ground into approximately 3 mm pieces and collected in a paper cartridge. The raw material was inserted into the extractor vessel which had been previously filled with ethanol.

The extractions were carried out in Batch Mode (BM) under the following conditions: 200 bar pressure, 80 °C temperature and 12 h running time. The CO${}_{2}$ was injected into the extractor until the desired operating pressure was reached. The extracts were blended together to produce a homogeneous ethanolic extract whose concentration was 91.775 ppm.

### 2.3. Supercritical Impregnation

**Figure 1:**
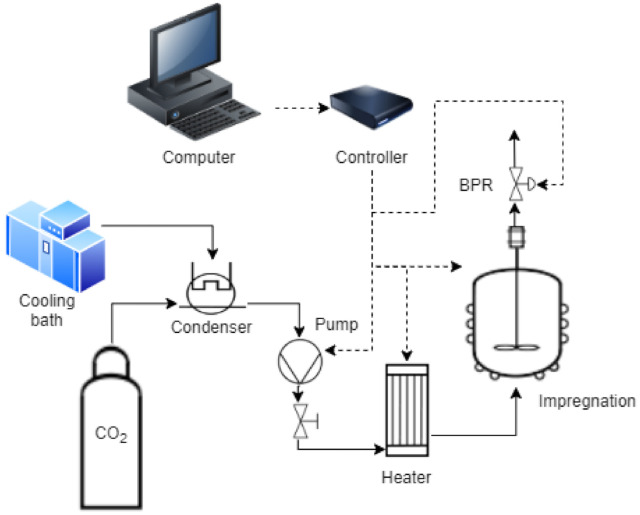
Flowchart of a supercritical impregnation. CO${}_{2}$ is condensed so that it can be pumped through the heater to the impregnation vessel where the impregnation takes place. The Back Pressure Regulator valve (BPR), the temperatures of vessel and heater, and CO${}_{2}$ flowrate are controlled via a specific software.

Impregnations were carried out into supercritical impregnation units (Figure 1) supplied by Thar Technologies (models SF100 and SF1000, Pittsburgh, PA, USA). Each one fitted with a CO${}_{2}$ reservoir tank, a high pressure pump with 50 g/min maximum flow rate, a thermal jacket, a magnetic agitation system, and an automatic back-pressure regulator valve to keep the system’s pressure.

The impregnation process was analyzed in two stages. In the first stage, a set of experiments was carried out in order to determine the best condition for the impregnation of the PLA filaments. A three-factor two-level factorial design was employed to determine optimal temperature, pressure and amount of MLE to be added to the vessel for the impregnations (Table 2). The response variables were the swelling of the PLA filament, the MLE impregnation loading and the antioxidant and antidenaturant capacities of the impregnated PLA filaments. Four 35 mm long and 1.6 mm wide PLA filaments were placed inside the SF100’s vessel by a steel structure to prevent any contact between the MLE and the filaments. Then, the reactor was turned on and once the operating temperature was reached, a 10 g/min CO${}_{2}$ flow rate was injected into the vessel until the preset pressure was reached. After 30 min of impregnation, a depressurization program was initialized at a rate of 30 bar/min.

All data obtained were processed by means of Statgraphics Centurion 18.

**Figure 2:**
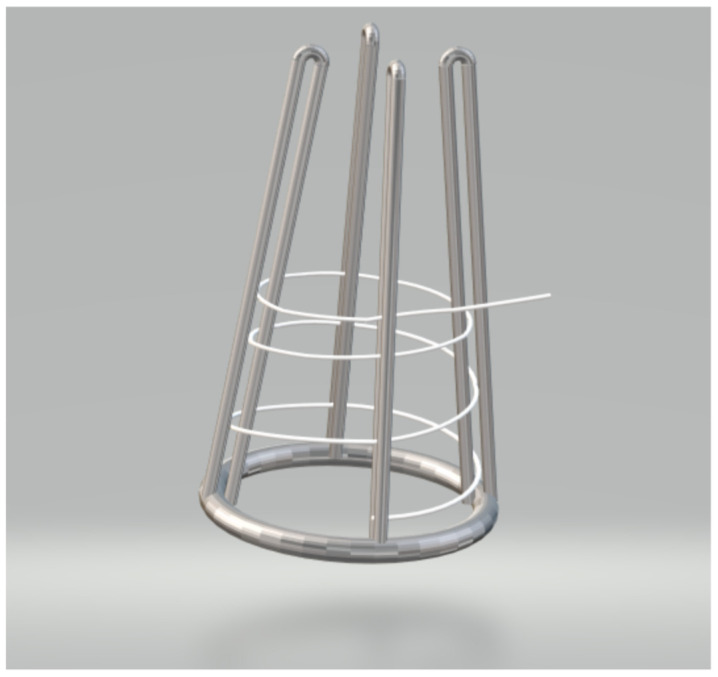
Approximate 3D representation of the structure used to support the 150 cm long PLA filament during its SCI.

At a second stage, once the best impregnation conditions had been established, the impregnation of a large filament, to be later on used for the 3D printing experiments, was performed by means of the SF1000 unit. A 150 cm long PLA filament was given a coiled shaped by means of a specific steel structure (Figure 2) and placed inside the vessel. The impregnation was conducted at 39 °C and 100 bar, using 30 mL of MLE. The run time was set to 24 h, so that time could be disregarded as an influencing factor. Pressurization and depressurization rates were kept at 10 g/min and 30 bar/min respectively for all the runs.

### 2.4. Scanning Electron Microscopy (SEM)

A Nova NanoSEM 450 scanning electron microscope was employed to detect any changes in the filaments’ surface. The samples were sputtered with a 10 µm coat of gold by means of a Cressington Sputter Coater 208 HR to increase their conductivity.

### 2.5. 3D Printing

**Figure 3:**
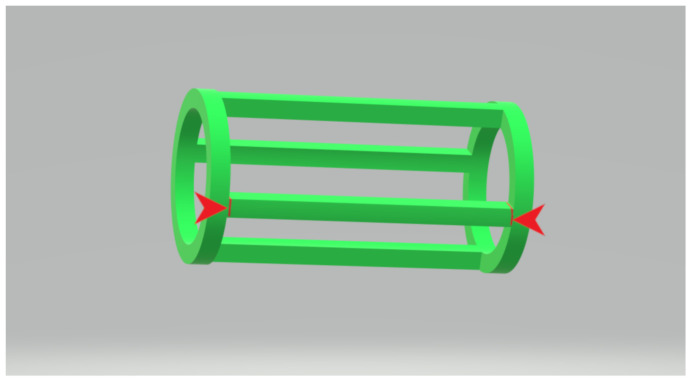
The elongated filament-like piece contained between the red marks was pulled apart from the structure to be used as experimental sample.

The 150 cm long filaments were used to perform a printing test using an FDM printer purchased from ANYCUBIC (model MEGA S, Shenzhen, China). The 3D model cylinder as shown in Figure 3 was taken from the website www.thingiverse.com (http://www.thingiverse.com/thing:3254734) by Russian_kwas is licensed under the Creative Commons attribution license (http://creativecommons.org/licenses/by/3.0/). The image in Figure 3 is derived from the original model to denote which parts of the printed model were used as samples. The fraction between the red marks was cut and used as individual sample, so 4 samples were obtained from each printing. The printing took place at 200 °C.

### 2.6. Foaming and Impregnation Loading

SSI consists of three phases, (i) dissolution of solute into the supercritical CO${}_{2}$ (scCO${}_{2}$), (ii) contact between polymer and supercritical solution, and (iii) diffusion of bioactive components into the swelled matrix of the polymer through the diffusion channels. Then, there is a depressurization phase to subtract all of the CO${}_{2}$ from the vessel and obtain a CO${}_{2}$ free polymer that is loaded with the bioactive compound [19,38]. When the polymer is saturated with CO${}_{2}$ under steady temperature and pressure conditions, and depressurization occurs abruptly, structural modifications can occur, especially when the temperature is quite above the T${}_{g}$, when foaming occurs. However, when T${}_{g}$ is around the working conditions, the polymer can be still in the glassy state [39], although some volume variations can occur. To evaluate these mechanical changes, the percentage of volume increment or swelling of the filament according to the equation given below [40], where %S is the swelling percentage, *V* is filament volume after the impregnation, and $V_{0}$ is the original filament volume before being impregnated:

(1) $$\%S=\frac{V-V_{0}}{V_{0}}\cdot 100$$

The impregnation loadings of MLE onto the filaments was gauged by means of gravimetric analysis. Equation (2) was used, in which %L is the loading percentage, $w_{0}$ is the initial weight of the filament before impregnation, and *w* is the weight of the filament after impregnation:

(2) $$\%L=\frac{w-w_{0}}{w_{0}}\cdot 100$$

The weight values of the polymer after impregnation were taken 24 h after the process, in order to let the CO${}_{2}$ diffuse and consider only the weight difference caused by the MLE loading.

### 2.7. Impregnated Filaments Bioactivity

The antioxidant (AOC) and antidenaturant (ADC) capacity of the impregnated filaments were measured to determine their bioactivity. For this purpose, the reduction of DPPH [41] in the presence of the antioxidant compounds in the filaments’ matrix was measured through an spectrophotometric method. Thus, a dilution of DPPH in ethanol at $6\cdot 10^{-5}$ M was used. First of all, a calibration curve was generated by measuring the response of an MLE concentration within a specific range at the following concentrations: 1.56 μg/mL, 3.13 μg/mL, 6.25 μg/mL, 12.50 μg/mL, 25.00 μg/mL, 50.00 μg/mL. Then, 3.9 mL of DPPH were mixed in 0.1 mL of the MLE and, after 2 h incubation, the solution absorbance was determined by means of a Shimadzu UVmini-1240 spectrophotometer (Sydney, Australia). The antioxidant capacity of the MLE was calculated as the percentage of oxidation inhibition (%OI) according to the following equation:

(3) $$\%OI=\frac{A_{0}-A}{A_{0}}\cdot 100$$

where $A_{0}$ is the initial absorbance of the DPPH solution, and *A* is the absorbance of the sample after the 2 h incubation. The empirical Equation (4) was used to calculate the half-maximal inhibitory concentration (IC${}_{50}$), where %OI is the percentage of oxidation inhibition, and *C* is the concentration of MLE (μg/mL). Then, in order to determine the efficiency of the extract, its Antioxidant Activity Index (AAI) was also calculated using Equation (5) [42]:

(4) $$\%OI=-0.1801C^{2}+7.7076C-1.2726\;$$

(5) $$\%AAI=\frac{\mathrm{final}\;\mathrm{concentration}\;\mathrm{of}\;\mathrm{DPPH}(\mu g/mL)}{IC_{50}\left(\mu g/mL\right)}$$

This parameter is a standardized reference of the AOC capacity of a particular substance. Thus, when AAI < 0.5, AOC is poor; when AAI is between 0.5 and 1.0, the extract shows a moderate AOC. If AAI is between 1.0 and 2.0, a relatively high AOC is registered; and if AAI > 2.0, then the extract exhibits a very strong antioxidant capacity [43]. The AOCs of both non-printed impregnated samples (NPIS) and printed impregnated samples (PIS) were measured using the same procedure, but in the case of PIS, instead of 0.1 mL of MLE, 0.1 mL of ethanol was used. Thus, the filaments were submerged into the solution and incubated for 2 h. The absorbance was measured following the procedure previously described. Non-impregnated PLA was used as a measurement control in each case.

On the other hand, to determine the ADC of the samples, the capacity of the agent to prevent the denaturation of egg albumin was measured by spectrophotometry [44]. As the MLE had an ethanolic base, it was first dried out and then diluted in water to produce an aqueous mango leave extract. This change of phase prevents the possible denaturation of the proteins that could be caused by the presence of ethanol and that could interfere with ADC measurements. The test solution was then prepared by blending 0.2 mL of a 1% egg albumin solution with 2.8 mL of PBS at pH 6.3 and with 2 mL of MLE. Two incubations were then carried out; the first one took place for 15 minutes in an oven at a temperature of 37 ± 2 °C, and the second one was conducted for 5 minutes in a boiling bath, in which the temperature was 70 ± 2 °C. After the incubation and cooling of the tubes, the absorbance was measured at 660 nm. PBS was used as blank and a reaction medium formed by 0.2 mL egg albumin solution, 2.8 mL PBS and 2 mL distilled water was set as control.

Equation (6) was used to calculate the antidenaturant capacity of the extract (%ADC). $A_{c}$ represents the control sample’s absorbance, $A_{s}$ is the tested sample’s absorbance and $A_{b}$ is the blank’s absorbance:

(6) $$\%ADC=100-\%Inhibition=\frac{1-A_{c}}{A_{m}-A_{b}}\cdot 100$$

Similar to the calculations of AOC, IC${}_{50}$ for the ADC of the extract, the empirical Equation (7) was employed:

(7) %ADC=18.6786lnC−36.8432

where *C* is extract concentration (μg/mL).

The ADC of the PIS and NPIS samples was determined following the same method, i.e., by submerging the filaments in the reaction medium and favoring the diffusion of the MLE during incubation. The reaction medium consisted of 0.2 mL egg albumin solution, 2.8 mL PBS and 2 mL distilled water and the incubation was carried out as described above. The untreated PLA was used as sample control, although any bioactivity was detected.

### 2.8. MLE Release and Evaluation of Long-Term Bioactivity

The diffusion kinetics of the extract was conducted to determine the release of the active substance from the impregnated polymeric filaments, both before and after the printing process. Additionally, the filaments’ AOC and ADC were determined after a long-time incubation period. Approximately 20 mg of each, NPIS and PIS, were submerged into hermetically sealed vessels containing 10 mL PBS at pH 6.3. They were kept at 37 ± 2 °C inside an incubator. An aliquot was taken at regular intervals and measured at 275 nm, corresponding to the absorbance peak determined for the water-based MLE, even at low concentration. After their measurement, the aliquots were returned to the container to maintain the volume. A calibration curve was built to correlate absorbance at 275 nm with MLE concentration in the medium.

(8) Abs=0.0179C+0.0126

where *Abs* is the absorbance at 275 nm and *C* is the extract concentration (μg/mL).

The amount of MLE released into the medium could be determined as µg of MLE per 100 mg of PLA. The printing process can affect not only the eventual diffusion kinetics, but also the bioactivity of the printed polymer, since high temperatures have to be employed to fuse the polymer before printing. In order to study the variation of bioactivity, both, NPIS and PIS were put aside to determine if their AOC or ADC had improved after a prolonged incubation period. For this purpose, 85 mg PIS and NPIS samples were also saved at 37 ± 2 °C into hermetically sealed vessels filled with 5 mL of PBS at 6.3 pH for 9 days. A PBS-based solution of MLE was generated. From this solution, 4.8 mL were used for the ADC test and the remaining 0.2 mL were used for the AOC test. The AOC test was run as described in Section 2.7, but this time, instead of the 0.1 mL ethanol-based MLE solution, the solution was based on PBS. On the other hand, for the ADC tests, the 4.8 mL of PBS-based MLE solution were blended with 0.2 mL of egg albumin dissolution. The rest of the experiment was conducted according to the description in Section 2.7.

Statgraphics Centurion 18 was used for statistical analysis of the data generated.

## 3. Results and Discussion

### 3.1. Impregnation of PLA

#### 3.1.1. Swelling

**Figure 4:**
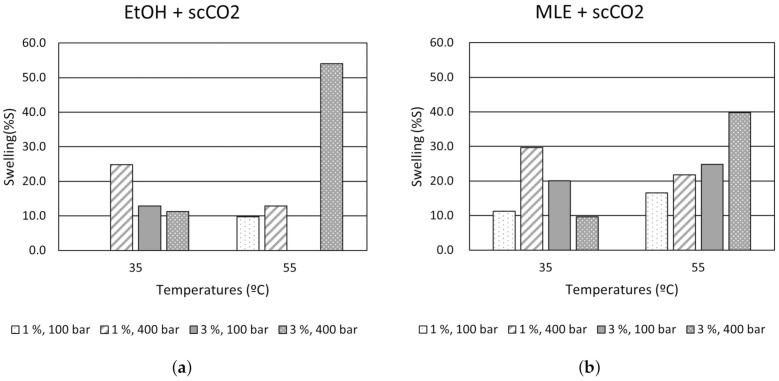
Evolution of swelling under the different impregnation conditions: (**a**) Swelling of the polymer filaments impregnated with just ethanol. (**b**) Swelling of the polymer filaments impregnated with MLE.

During impregnation, there is an increase in the free-volume of the polymer caused by its plasticization, which is known as swelling. This modification can cause a variation on the diameter and length of the filament. Since it is fed into the printer, the swelling effect may render them unsuitable for printing purposes. Thus, the modification of the polymer volume after impregnation was determined evaluating the influence of the different operational conditions. Figure 4 displays the polymeric filaments’ swelling progression when using ethanol (Figure 4a) and MLE (Figure 4b). At low temperature (35 °C) and low pressure (100 bar), the swelling percentage of the polymeric filaments increased when the amount of ethanol was increased from 1% to 3%, observing a different trend at 400 bar. On the contrary, the behavior was the opposite at high temperature (55 °C), highlighting the lower values of swelling at 100 bar and the significantly high values at 400 bar when the proportion of the co-solvent volume increases (from 1% to 3%).

Under supercritical conditions, the interaction between the CO${}_{2}$ and the ethanol disrupts any predictable solvent’s density, as it was reported by Pöhler and Kiran [45]. This phenomenon could explain why unpredicted swelling may occur. Under 400 bar and with 3% co-solvent, swelling reaches its highest values. The presence of an organic solvent, such as ethanol, promotes PLA plasticizing, so its internal structure’s chains gain mobility and a larger swelling takes place. This phenomenon is favored with high pressures, since not only the sorption of CO${}_{2}$ is higher, but also the diffusion is facilitated, which is determining in the functionalization of polymers with active compounds [46]. This behavior could explain why PLA experienced such a great swelling under 55 °C and 400 bar with 3% co-solvent, both using just ethanol and MLE. In fact, a general rise in swelling values is observed when the impregnations were carry out with MLE in comparison with the results obtained when using only the co-solvent (Figure 4b). While at 35 °C, the results follow the same trend, which is in agreement with other authors [18,47], at high temperature (55 °C), swelling increased when conditions were more extreme. It must be underlined that all the filament samples impregnated at 400 bar and 55 °C at 3% co-solvent of the total vessel volume, presented some damages, such as end-to-end longitudinal slits. Therefore, polymeric integrity is a factor to bear in mind.

#### 3.1.2. Impregnation Loading

**Figure 5:**
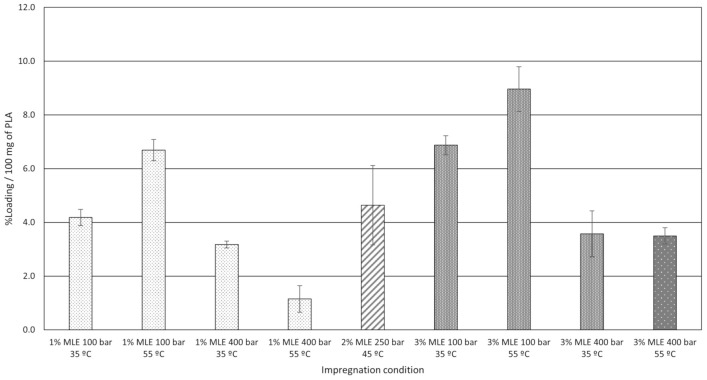
MLE loading at each set of impregnation conditions.

Figure 5 depicts the MLE loadings onto the samples, which has been calculated gravimetrically. It must be taken into account that, because of supercritical impregnation is a two-way mass transfer process, at the same time MLE is being absorbed into the polymer, additives and a low molecular weight compound could be dragged out by CO${}_{2}$. Gauging the amount of MLE impregnated in a polymer by a gravimetric method does not consider the aforesaid losses of mass, hence some deviations may occur on the loading reported with respect the real MLE amount incorporated into the polymer matrix. However, clear tendencies of the results can be observed, leading to valid conclusions.

The explanation of the behavior of the system scCO${}_{2}$/PLA/MLE fits with the statements described by Rojas et al. [46]. When a greater volume of MLE is added, the incorporation to the PLA filaments is favored for both pressure conditions (100 bar and 400 bar) since the concentration gradient between the CO${}_{2}$ phase and polymer increases. This would also indicate that even using 3% of MLE, the miscibility of the extract on the scCO${}_{2}$ seems favored and the compound could still not be in excess. However, the variation of the solubility depending on the pressure/temperature conditions provide different loadings. Under low pressure values (100 bar), the loading seem to be favored by temperature, as reported by Sugiura et al. [48]. The increase in temperature decrease the CO${}_{2}$ density, which provides a higher concentration of the compound in the supercritical phase, leading to a higher impregnation. In this sense, the loading achieved the highest value at 3% MLE of vessel volume, 100 bar and 55 °C. On the other hand, the higher CO${}_{2}$ density at higher pressures causes a better affinity of the compounds to the supercritical phase, leading to lower impregnation values. Similar results were reported by Milovanovick et al. [47]. In their research, they impregnated PLA and poly (lactic-co-glycolic acid) (PLGA) spheres under certain conditions, then, new impregnations were executed at a higher pressure in order to determine how a greater scCO${}_{2}$ density could affect its capacity to carry the solute into the polymer. As aforementioned, integrity is an issue to bear in mind when impregnation takes place at 400 bar and 55 °C with 3% MLE of vessel volume. This is why it has been set apart from the rest of the conditions in Figure 5.

**Figure 6:**
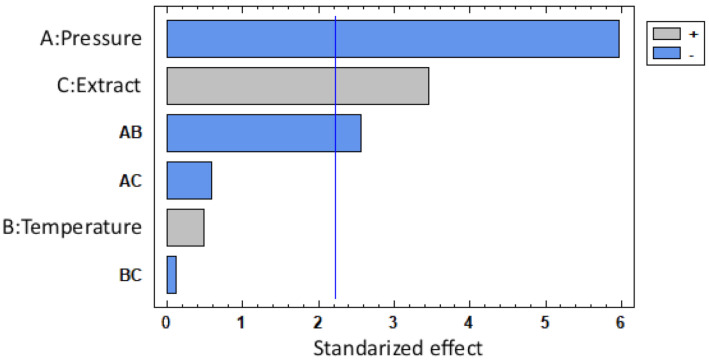
Pareto chart showing MLE loading.

In this sense, there is a high correlation between the MLE loading and the operating pressure. The Pareto chart in Figure 6 shows the standardized effects for each variable for loading, where the negative influence of pressure can be confirmed. The interaction between temperature and pressure has proven to have a significant effect on MLE loadings, probably due to the correlation between scCO${}_{2}$ density and each one of these two variables.

Figure 6 also confirms that the amount of extract in the vessel during the impregnation process has a direct positive effect on the loading. This would confirm that the higher the amount of active compound in the scCO${}_{2}$ is, the larger the amount of MLE that becomes impregnated onto the matrix.

### 3.2. Scanning Electron Microscopy (SEM)

**Figure 7:**
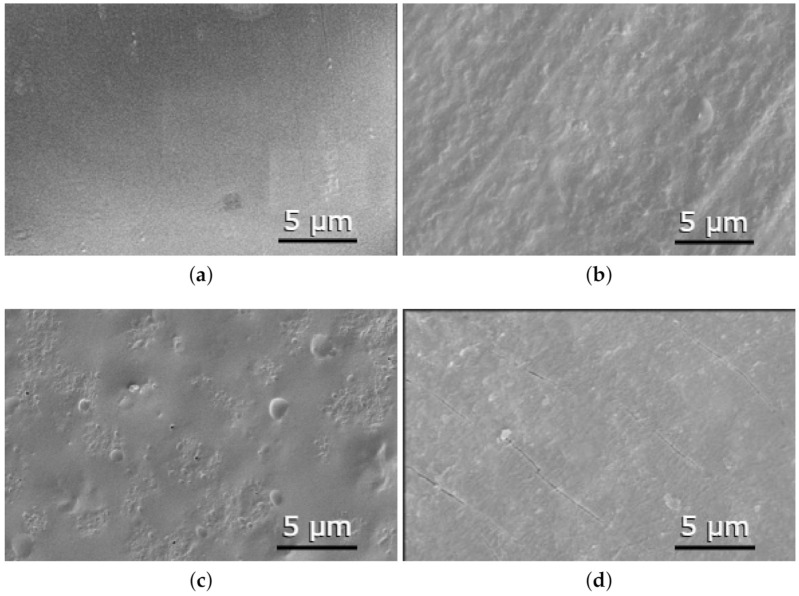
Scanning electron microscopy of samples: (**a**) non-impregnated PLA (5000×), (**b**) impregnated at 3% of ethanol, P = 100 bar, T = 35 °C (5000×), (**c**) impregnated at 1% of MLE, P = 100 bar, T = 35 °C (5000×), (**d**) impregnated at 3% of MLE, P = 100 bar, T = 35 °C (5000×).

The samples with the most representative results, as well as a PLA control sample were observed under the microscope to detect any differences on the polymer’s surfaces before and after their impregnation with ethanol and with MLE. The samples selected for this step were non-impregnated filaments, filaments impregnated at 100 bar and 35 °C with just ethanol at 1% and 3% and with MLE at 1% and 3%. The appearance of those filaments’ surfaces are depicted in Figure 7.

Non-impregnated PLA shows a smooth and regular surface without any scratches, compared with the rough surface corresponding to a sample impregnated at 1% ethanol, 100 bar and 35 °C. This picture evidences that PLA is affected by the impregnation process, and suggests that not only the polymer surface has been modified, but also its internal structure. Figure 7c depicts an irregular surface with bulges, probably due to particles lodged inside the polymer as well as some surface pores resulting from the swelling of the polymer in the presence of the extract (Figure 4b). Finally, in the sample impregnated at 100 bar and 35 °C with 3% of MLE (Figure 7d), surface cracks can be observed, probably due to the higher swelling percentage of PLA at those conditions. This picture makes us think that the internal structure of the PLA filament has been fairly altered. Such alterations could imply the appearance of critical mechanical dysfunctions that could affect the subsequent performance of the polymer when used for the manufacturing of medical devices.

### 3.3. Bioactivity of the Impregnated PLA

#### 3.3.1. Antioxidant Activity

**Figure 8:**
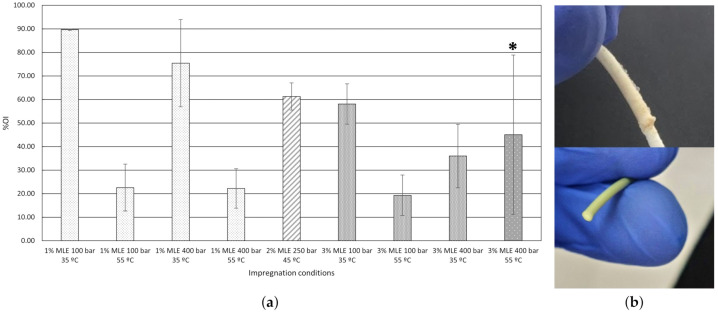
(**a**) Percentage of oxidation inhibition (%OI) displayed by MLE-impregnated PLA filaments. * Structural damages. (**b**) Examples of damaged filament impregnated at 400 bar and 55 °C with 3% of MLE (top) and a representative sample of a non-damaged filament.

The extract has proved to be a very strong antioxidant agent, as it can be seen by the IC${}_{50}$ and AAI values, 8.24 μg/mL and 2.80 μg DPPH/μg extract, respectively, which were calculated using Equations (4) and (5). Besides, impregnation efficiently transferred those properties to the polymer given the filaments’ high values of %OI, as can be seen in Figure 8a. The filament samples impregnated under low pressure and temperature (100 bar and 35 °C) conditions reached higher antioxidant capacity values. In fact, the samples impregnated with 1% MLE of vessel volume and under 100 bar and 35 °C reached 89.63% oxidation inhibitory capacity. Goñi et al. [49] performed an SSI under similar conditions (150 bar, 45 °C and 50 bar/min depressurization rate), on Linear Low Density Polyethylene (LLDPE) films using eugenol as the active compound where the tested samples exhibited 81% oxidation inhibitory capacity. In their study, according to the SSI results, the film samples that were depressurized at a rate of 5 bar/min also reached AOC values as high as 80%. In a similar study by Franco et al. [50], monolayer and multilayer films of terephthalate (PET) and polypropylene (PP) were impregnated by SSI at 170 bar and 40 °C, with α-tocopherol and all of the film configurations displayed an oxidation inhibitory capacity of approximately 92%. All of the above-mentioned SSIs were conducted at temperatures under 50 °C and functionalized polymers with a great AOC were obtained in all the cases. However, the impregnations that were carried out in this study at 55 °C showed lower oxidation inhibitory properties.

**Figure 9:**
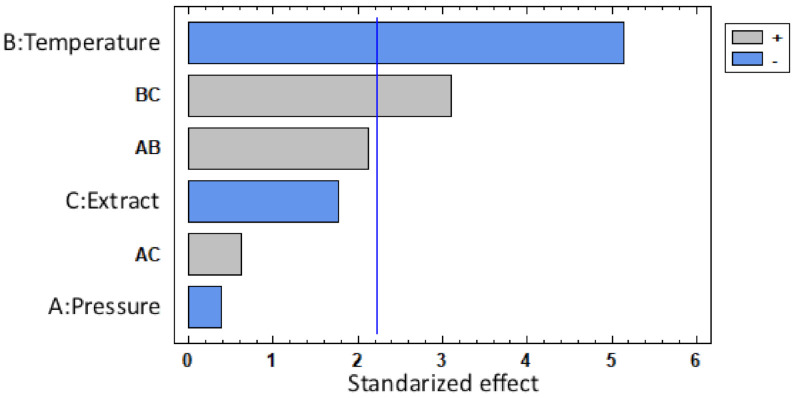
Pareto chart showing AOC values of the MLE-impregnated PLA filaments.

Attending the results collected in Figure 5, bioactivity and loading are not directly related, since the highest conditions loaded (100 bar and 3% at both temperatures), are not those with the highest AOC. This behavior has been previously observed when using a complex matrix such as MLE as an active matter. The different conditions favor a better dissolution of certain compounds at different operational conditions, leading to variations in the filament composition that eventually is reflected in the bioactivity. This selectivity on impregnation was proven previously by Cejudo et al. both in the impregnation of olive leaf extract in PET/PP and mango leaf extract in nanofibrillated composites [51,52]. As it can be seen in Figure 9, temperature is the variable with the most significant influence with regard to the AOC shown by the impregnated polymers. This higher AOC could be related to the affinity between the solute and the scCO${}_{2}$. It is well known that, to a large extent, the solubility of the bioactive compound depends on the solvent density. Thus, since scCO${}_{2}$ becomes denser as the temperature drops, some compounds would not dissolve well in the supercritical solvent, which in turn, would result in a poorer mass transfer. Having said this, impregnation loadings may also be related to the difference between the bioactive compound’s affinity with the polymer in comparison with its affinity with the solvent. Thus, at high temperatures, the solute’s affinity with the solvent could be higher than its affinity with the polymeric matrix, which in turn, would favor the solute to be dragged out of the vessel together with the CO${}_{2}$ if a high depressurization rate is applied [13]. It should be noted that, although pressure has not been considered as a major variable, when impregnations were carried out at 100 bar, the functionalized polymer filaments exhibited higher AOC values than those processed under higher pressure conditions (400 bar). Nevertheless, it is also true that when the filaments were impregnated at 400 bar and 55 °C with 3% MLE, their structure was more severely altered (Figure 8b), which results in a low reproducibility of the analysis.

#### 3.3.2. Antidenaturant Activity

Table 3 displays the percentage of ADC (%ADC) shown by the impregnated filaments processed under each set of conditions. Since the ADC is performed by the extract, the concentration of MLE released into the medium ([MLE]) has been indicated in the second column of the table according to Equation (7). The proportion of MLE released (%MLE released) from the polymer is also shown in the table as the amount of MLE released into the medium divided by the total MLE loading of each sample. The proportion of MLE released provides information on the amount of the loaded extract that migrates from the polymer into the medium. Since ADC was only measured 20 min after completing the incubation period (15 min from the first incubation and 5 min from the second one), MLE migration into the medium only reached up to 2% of the total MLE loading in each sample, which implies that the measurement of the ADC corresponds just to a part of the extract that had been impregnated into the polymer.

**Figure 10:**
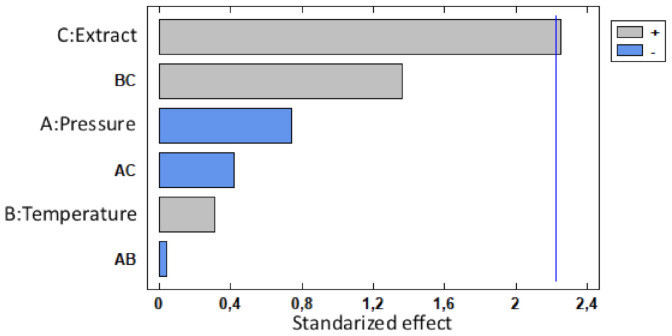
Pareto chart representing the ADC of the MLE-impregnated PLA.

The best ADC responses are achieved by the samples from SSI conditions of 3% MLE and 100 bar (22.78% for 35 °C and 18.86% for 55 °C), both standing for the most swelled and loaded filaments. This result is in agreement with the statistical analysis, since only the amount of extract poured into the vessel should be considered as relevant regarding the ADC level shown by the filaments after their impregnation, as shown in Figure 10. Even though swelling was not considered a factor but rather a response, it may still influence ADC, since it is closely related to MLE release. When the polymer swells it becomes more porous, so that the MLE diffusion is facilitated and a greater amount of the extract reaches the test medium.

The absence of ADC in the sample produced at 400 bar and 55 °C 1% MLE condition, suggests that temperature may have had an adverse effect on ADC. A similar issue occurs with the sample impregnated at 100 bar, 55 °C using 3% MLE, which exhibits a lower ADC in comparison with that of sample impregnated at 100 bar and 35 °C with 1% of MLE. However, as can be seen in Figure 10, only the amount of extract poured into the vessel resulted in a significant change regarding ADC values shown by the filaments after their impregnation.

Bearing in mind the ADC shown by the impregnated filaments, it is interesting to focus on the results for IC${}_{50}$ from Equation (7), which should represent how effective this extract is to prevent the denaturation of the proteins. Since the IC${}_{50}$ is 104.50 μg/mL, we can compare this value against the efficacy of other extracts as determined by a similar methodology. The MLE showed a moderated ADC when compared against the extract obtained from *Oryza sativa* (rice) [44] or *Caffea arabica* (coffee) [53], which showed greater inhibition levels of protein denaturation. These extracts inhibited protein denaturation up to 75% at a concentration of 100 µg/mL, and up to 50% at 40 μg/mL, respectively. However, MLE performed better than the extract obtained from *Murraya koenigi* (curry) [54], which showed a 52.38% inhibition of protein denaturation at a concentration of 250 μg/mL, which is more than double the concentration at which MLE reached a similar ADC level.

Due to the alterations suffered by the filaments, when processed under 400 bar and 55 °C with 3% MLE of the vessel volume, it is difficult to determine which is the real effect that high pressure has on ADC. The end-to-end deep groove, that each filament presented when impregnated under the mentioned condition, affects mass transfer during the impregnation process and MLE release during the ADC tests that have been carried out in our study.

### 3.4. Filament Production for 3D Printing

By applying multiple response optimization to the best results, a 150 cm long filament was produced. The optimal impregnation conditions were established by maximizing a combined desirability function, which was obtained from the individual optimization of each response variable. The response variables considered for optimization purposes were loading, bioactivity and swelling, where swelling should present its minimum value. According to the results included in Table 3, the resulting optimal conditions—100 bar at 39 °C with 3% MLE of the vessel volume—were applied to impregnate 150 cm long filaments to be later on used for the 3D printing tests. The 150 cm long filaments were impregnated under the established optimal conditions indicated in Table 4. The impregnation time was increased to 24 h, in order to guarantee to establish the thermodynamic equilibrium of the samples. Once impregnated, each filament was fed into the 3D printer, and a preset model was generated. The printed samples were subjected to release and bioactivity tests and the results were compared against those of the NPIS.

The impregnation for the 150 cm long filament was carried out under the optimized conditions gathered on Table 4. The impregnation time was increased to 24 h, in order to guarantee the complete impregnation of the sample. Once impregnated, this filament was introduced in the 3D printer, and the selected model was manufactured. The printed impregnated samples were used in the release studies and the bioactivity tests; then, the results were compared with those of the non-printed impregnated samples.

**Figure 11:**
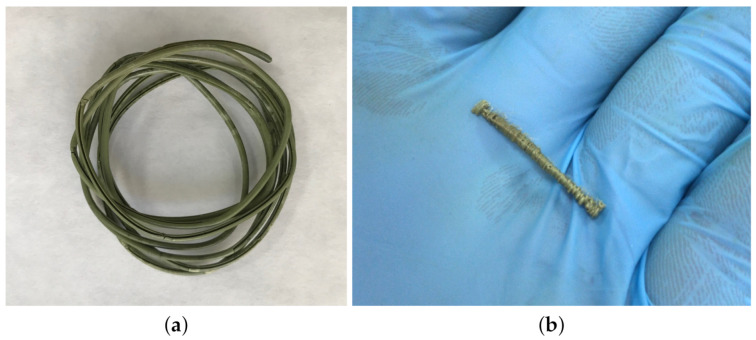
Pictures of the polymeric samples used for the evaluation of ADC and AOC (**a**) 150 cm long PLA filament after impregnation. (**b**) Sample used for the biocapacity tests of printed PLA.

Figure 11a displays a 150 cm long filament after its impregnation, whilst Figure 11b shows one of the printed samples used for bioactivity evaluation.

#### 3.4.1. MLE Release Study of the Impregnated and Printed Filaments

**Figure 12:**
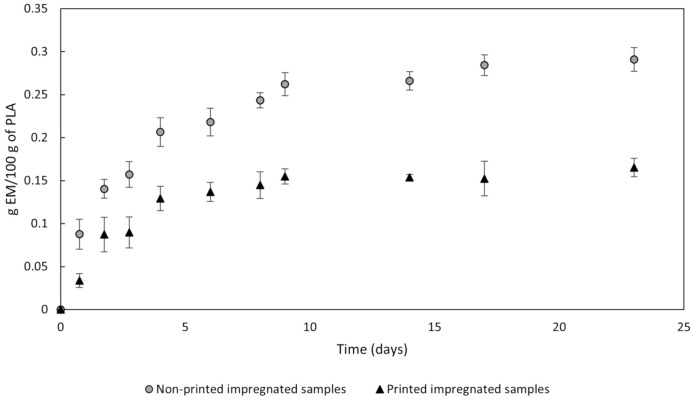
MLE release profile of non-printed and printed samples.

Due to the structural disruptions caused on the polymer during printing, the internal structure of the filaments resulting from the fused deposition printing process differed largely from that of the filaments used to feed the printer. Therefore, a comparison between both structures was required. PLA prints are generated by rapid layer over layer deposition of fused material which coalesces and constitutes a compact and dense object with a limited number of small size pores [55]. This process affects the capacity of the MLE to migrate from inside the polymer into the medium, which in turn influences the bioactive capacity of the polymer. Figure 12 displays the data corresponding to the MLE released from the printed and non-printed samples into a PBS medium. It can be seen that the liberation of the MLE from the printed samples decreased with respect to the non-printed filaments. The plateau of the curves shows a different amount of MLE extract released at the equilibrium condition. Considering that the samples analyzed come from the same impregnation experiment, and therefore have the same loading, the differences in the signal could be related either to changes in the microscopic structure of the polymer, that change the kinetics of the migration, and a certain compound degradation due to the high temperatures employed during printing. The lower slope in the curve of the printed PLA filaments shows that the structure of PLA changed drastically during printing, affecting probably the crystallinity and mechanical properties, and leading to a decrease in the diffusion coefficient of the MLE. However, to confirm these modifications, further mechanical analysis should be done. Since the final objective of this study is to determine the suitability of 3D printing for the production of biomedical devices that allow a slow administration of an active substance over a relatively long period of time, this progressive liberation is actually a rather convenient outcome. In order to verify the remaining bioactivity of the MLE after printing, it is necessary to analyze the bioactivity of the product released. If this activity is proportional to the concentration of the MLE in the medium, the effect of this decrease in the concentration should be attributed to the modification of the structure of the PLA, and therefore, in the diffusion coefficient.

#### 3.4.2. Bioactivity of the Printed Bioactive PLA

Regarding to the bioactivity of the PIS, they were tested and compared against NPIS as described in Section 2.7 and Section 2.8. Table 5 presents the bioactivity data—ADC and AOC—by the four types of filaments, i.e., NPIS and PIS, as well as non-incubated filaments and filaments after 9-day incubation in PBS, where the plateau of the curve is reached in each condition (Figure 12). The ADC of the non-incubated samples went down from 11.07% to a null capacity to inhibit denaturation after being subjected to the printing process. On the other hand, their AOC also dropped, although not so drastically. These results were in agreement with the alteration of the diffusion channels that takes place over the printing process, as previously described in Section 3.4.1.

However, bioactivity differences become more obvious when longer incubation times are applied. In fact, in an aqueous medium such as PBS, the hydrolysis of the PLA ester bonds takes place while carboxyl and hydroxyl groups are generated. The former one promotes the autocatalysis of the PLA, so that the polymer gradually degrades, starting from the surface and progressing towards its internal structure. As this happens, the liquid medium diffuses into the polymer and new micro-cavities appear that promote further release of the compounds [56,57]. Subsequently, larger amounts of the extract migrate outside the polymer and a stronger effect from the extract biocapabilities is to be expected. First, this can be noticed by the great raise of ADC shown for the incubated samples. NPIS performed treble the percentage of denaturation inhibition after incubation, compared to the non-incubated samples. Additionally, PIS obtains an ADC of 23.5% opposite the null ADC obtained from non-incubated PIS.

These results are rather encouraging, since a PLA-implanted device could exhibit this specific bioactivity for a prolonged period of time. On the contrary, AOC fell from approximately 90% for non-incubated NPIS to a scarce 10% after incubation, and the same goes for the PIS, which dropped from around 80% to 2.5% after the 9-day incubation. This loss of AOC must be caused by the hydrolytic degradation of PLA in aqueous solutions as a consequence of the hydroxyl groups that are generated and act as reactive oxygen species. This must the reason why the AOC test resulted in such low values, even though filaments were kept in hermetically sealed vessels and a greater amount of MLE was released from the polymer. In addition, it has to be considered that the evaluation of AOC of non-incubated samples was realized in the DPPH ethanolic solution, and since ethanol favorably dissolves MLE, a greater amount of extract had been released, showing a better AOC. Considering the results, the high temperatures employed in the printing process seem not substantially affect the bioactivity of the polymer, since it is maintained to a great extent. Probably, the decrease in the values obtained is more likely due to the change on the polymer conformation, which affects the diffusion kinetics, as was stated in Section 3.4.1.

## 4. Conclusions

PLA filaments impregnated with a bioactive substance were produced to feed a 3D printer. The influence of pressure, temperature and amount of extract to produce the feeding filaments were determined and optimized according to the response variables swelling, loading and bioactive capacity of the PLA filaments before feeding them into the printer. The optimum values of these variables after the experimental design analysis were 100 bar, 39 °C and 3% of MLE ethanolic solution in the vessel. This impregnation condition provides filaments with a greater amount of extract highly bioactive, in terms of antioxidant and antidenaturant capacities, with low physical modifications. Further, those capacities have been proven to remain after the printing of PLA, which has not been previously assessed.

3D printing is a rather recent technology that has found its practical use in many fields such as medicine. It is not hard to imagine that functionalized medical devices will soon be produced in a single-step process, reducing costs and time enhancing surgery preparation and patient outcome. Such devices would provide a new form of drug administration thanks to the many advantages of additive manufacturing. Many forms of implants such as stents or bone prostheses could be made out of bioactive polymers, which would contribute with extract-impregnated derived properties to a more complete medical treatment. Even though, further research should be carried out in vivo, the data obtained in our study suggest that PLA is an excellent material to administer antidenaturant compounds into the patient’s body. On the other hand, the consequences of the PLA hydrolysis oxidative effect over antioxidant activity in an aqueous medium and in vivo must be studied, since it is possible that the patient would not benefit from the antioxidant properties of the device. Nevertheless, the results from our study have confirmed that polymers can be provided with bioactive properties directly from natural extracts, and given that medical devices are precise implants, thorough research should be conducted on the influence of the impregnation process variables on the mechanical characteristics of the polymeric structures for a more precise manufacturing and development of polymeric medical devices.

## Figures and Tables

**Table 1:** Thermal properties of the applied PLA material.

Property	Value (°C)	Standard Test
Heat distrosion temperature	56	ISO 75/B
Melting temperature	145–160	ASTM D3418
Glass transition temperature	56–64	ASTM D3418

**Table 2:** Analyzed variables for the impregnation process.

Variable	Value
Temperature (°C)	35, 45 *, 55
Pressure (atm)	100, 250 *, 400
Amount of MLE (% vessel volume)	1, 2 *, 3

* Average point for statistical analysis.

**Table 3:** Migration of MLE from impregnated filaments; percentage of ADC, concentration of MLE released into the medium and proportion of MLE released from each sample.

Impregnation Condition	%ADC	[MLE] (μg/mL)	%MLE Released
1% MLE, 100 bar, 35 °C	5.90 ± 4.81	10.02 ± 2.55	1.36 ± 0.54
1% MLE, 100 bar, 55 °C	13.33 ± 0.00	14.68 ± 0.00	1.23 ± 0.10
1% MLE, 400 bar, 35 °C	15.93 ± 4.43	17.10 ± 3.99	2.81 ± 0.53
1% MLE, 400 bar, 55 °C	n.d.	7.19 ± 0.00	10.04 ± 11.21
2% MLE, 250 bar, 45 °C	4.30 ± 2.94	9.11 ± 1.43	1.27 ± 0.25
3% MLE, 100 bar, 35 °C	22.78 ± 1.92	24.40 ± 2.50	1.93 ± 0.26
3% MLE, 100 bar, 55 °C	18.86 ± 3.10	19.87 ± 3.28	1.54 ± 0.31
3% MLE, 400 bar, 35 °C	6.67 ± 0.00	10.27 ± 0.00	1.39 ± 0.24
3% MLE, 400 bar, 55 °C	25.00 ± 0.00	27.41 ± 0.00	4.66 ± 0.33

**Table 4:** Low and high values of explanatory variables and optimized condition setting.

Factor	Low	High	Optimum
Pressure	100.0	400.0	100.0
Temperature	35.0	55.0	38.7
Extract	1.0	3.0	3.0

**Table 5:** Bioactivity of the NPIS and PIS before and after a 9-day incubation in PBS.

	%ADC_*non*−*inc*_	%AOC_*inc*_	%AOC_*non*−*inc*_	%AOC_*inc*_
Non-printed PLA	11.1 ± 2.8	30.1 ± 6.5	88.1 ± 1.9	9.9 ± 1.1
Printed PLA	n.d.	23.5 ± 2.2	79.1 ± 16.2	2.5 ± 2.5

*inc* denotes incubated.

## Data Availability

All the data presented in this study are available in this article.

## Sample Availability

Samples of the PLA impregnated are available from the authors.
